# Pulmonary Vein Compression After Implantation of a Left Atrial Appendage Occluder: Presentation and Discussion of a Case

**DOI:** 10.1016/s0972-6292(16)30775-6

**Published:** 2014-07-15

**Authors:** Maryam Ayati, Feifan Ouyang, KH Kuck

**Affiliations:** Department of cardiology, Ak St. Georg, Hamburg, Germany

**Keywords:** LAA occluder, Pulmonary vein stenosis, RF ablation, MDCT

## Introduction

Atrial fibrillation (AF) is the most common sustained cardiac arrhythmia which it occurs in 1-2% of the general population.[1] Over 6 million Europeans are suffering from this arrhythmia, and its prevalence is estimated to be at least double in the next 50 years. The prevalence of AF increases with age, and is from 0.5% at 40-50 years to 5-15% at 80 years.[2] Clinically, AF is associated with increased rates of death, stroke and other thromboembolic events, heart failure and hospitalizations, reduced quality of life and left ventricular dysfunction. In ischemic-related stroke and AF patients, emboli are dominantly from the left atrial appendage (LAA). Clinical studies have demonstrated that implantation of a LAA occluder can prevent ischemic stroke and avoid anticoagulation therapy in the patients with high-risk bleeding [3-5]. Currently, two different LAA occluders are available. However, it is still unknown whether the device can lead to depression or stenosis in PV diameter. In this article, a compression in the left inferior pulmonary vein after implantation of a LAA occluder is reported.

## Case report

**Figure 1:**
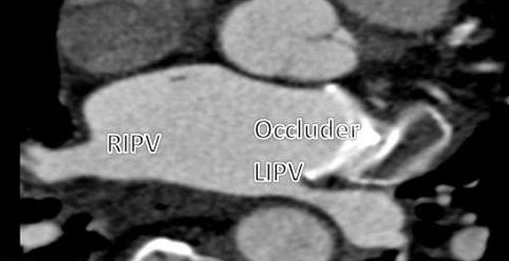
LAA occluder which narrowed the LIPV ostium

A 76 year old woman with primary hypertension was referred to our hospital for catheter ablation of persistent AF. She was suffering from exertional dyspnoe since two years ago which became more sever since the last 3 months. Persistent AF was initially diagnosed due to ischemic stroke on December 2011. Due to high risk of bleeding with anticoagulation, an Amplatzer LAA occluder (puck and disc type, AGA Medical Corp., Minneapolis, MN) was implanted in another hospital. Transesophageal echocardiography (TEE) was performed to exclude LAA thrombus before ablation procedure which confirmed that the LAA occluder was in the LAA. An angio CT of pulmonary veins was performed which showed three and two pulmonary veins in the right and left side, respectively. Compressed LIPV was suspected with an oval shape appearance in short axis (8 mm in CT angio), the other veins were in normal sizes (Figure 1).

**Figure 2:**
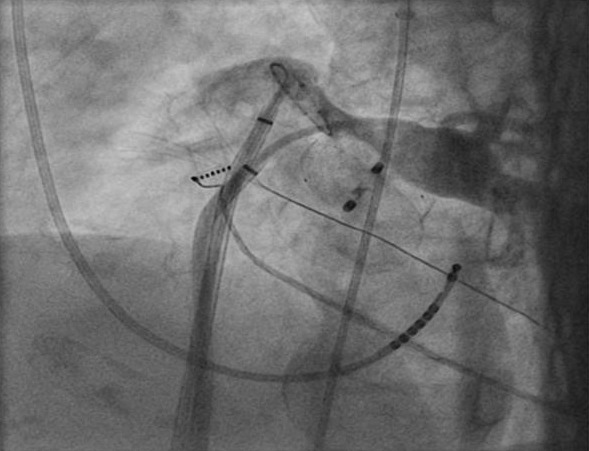
Angiography of LIPV shows the compression and narrowing of ostium

During the ablation study, stable sinus rhythm was at the beginning of the procedure. Two 8.5 F SL1 sheaths (St. Jude Medical, Inc., MN, USA) were advanced to the left atrium (LA) by a modified Brockenbrough Technique. After transseptal catheterization, intravenous heparin was administered to maintain an activated clotting time of 250 to 300 seconds. Additionally, continuous infusions of heparinized saline were connected to the transseptal sheaths (flow rate of 10 ml/h) to avoid thrombus formation or air embolism. Electroanatomical mapping was performed with a 3.5-mm-tip catheter (ThermoCool, Biosense-Webster, Diamond Bar, CA, USA) during SR by using CARTO 3 system (Biosense-Webstar, CA, USA). Mapping at the ridge between the left inferior pulmonary vein (LIPV) and LAA in anterior part showed significant decrease in impedance (about 70-80 Ohm), which suggested the catheter contact to the metal device (Figure 2).

**Figure 3:**
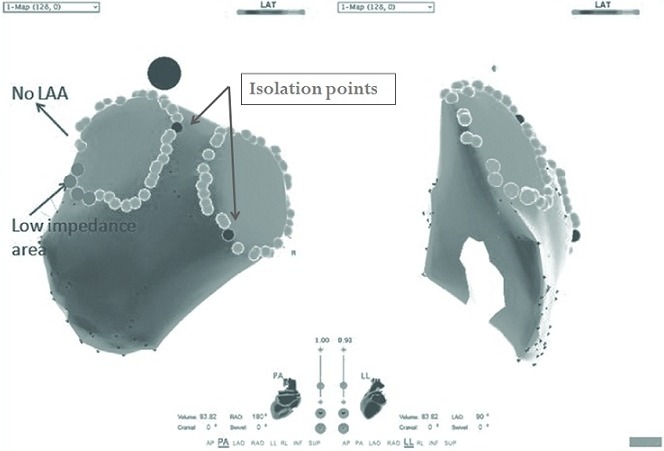
CARTO imaged showed the lower impedance in blue point area (80 Ohm in camparison with 110 in other parts), so the RF wasn't delivered in this part

Selective PV angiography showed that the right sided and left superior pulmonary veins (LSPV) had normal sizes but the LIPV was compressed with the atrial part of the occluder (the diameter of the vein in distal part just before the ostium was around 50 percent smaller than that of its proximal part (Figure 3).

There was no pressure gradient by pulling back from the distal to the LA with a multipurpose catheter 7F (St Jude Medical, USA). Circumferential radiofrequency (RF) lesions were delivered at the area with normal impedance that resulted in the complete PV isolation. Therefore, RF ablation was not necessary to deliver at the area with low impedance. The patient discharged home after 24 hours holter monitoring (stable SR) with Dabigatran for 3 months. Dyspnoe was slightly better.

## Discussion

Pulmonary vein stenosis (PVs) is a rare but significant complication after RF ablation of atrial fibrillation that poses a diagnostic challenge for the clinicians and can happen in 1-3 percent of cases with post RF ablation of pulmonary veins [6,7]. The incidence depends on the ablation strategy and the use of the energy. Generally, it can be diagnosed by noninvasive or invasive image techniques. So far, multidetection computer tomography (MDCT), TEE and magnetic resonance imaging (MRI) are the best ways to show the anatomy and morphology of LAA which can be used preprocedural and intraprocedural styles to guide the correct placement of occluder implantation [9,10].

A recent study has shown that LAA morphology is classified as Chicken Wing (18.3%), WindSock (46.7%), Cauliflower (29.1%) and Cactus type (5.9%) [9,10]. Interestingly, the Chicken Wing LAA morphology has demonstrated higher potentials for thromboembolism compared to other morphologies [11]. In patients with high risk of thromboembolization and bleeding, the LAA occluder is an alternative approach to reduce potential embolism and avoid the bleeding due to anticoagulant. In clinical practices, there are two different LAA occluder devices {WATCHMAN devices (Atritech Inc., Plymouth, Minnesota)} and {Amplatzer Cardiac Plug (ACP) devices (AGA Medical Corp., Minneapolis, MN)}. So far, there is very limited information about LAA Occluders' effect on the left-sided PVs. Anatomically the left-sided PVs is located posterior to the LAA with a narrow ridge between them. Generally the ridge is about 5-7 mm in diameter. The LAA occluder may theoretically have adverse effects on the left-sided PV. To our knowledge, this is the first case report of such a complication after implanting an LAA occluder in literature; however, the incidence of pulmonary vein stenosis (PVs) and natural progression over time are still unknown. Therefore, it should be taken into consideration that implantation of ACP may have an influence on the left-sided PVs.

## Conclusion

With increasing the frequency of RF ablation, one should consider the risk of PV Stenosis and control the patient after procedure to diagnose the symptoms as soon as possible. Recently, LAA occlusion is steadily used in clinical practice to prevent thromboembolism and reduce the bleeding risk in high-risk patients. In the present case, PVs was identified after LAA occlusion implantation and was diagnosed with preprocedural evaluation via MDCT, intraprocedural TEE and invasive PV angiography. This patient was also symptomatic (had exertional dyspnoe) which became more sever after LAA Occluder implantation. In addition, we couldn't identify the exact size of Occluder, but we guessed an oversized occluder should have been implanted. Also, complete PV isolation was successfully performed which was guided by PV angiography and impedance mapping. This may provide new ablation strategy to avoid unnecessary and unintended ablation within the PVs. Finally, systematic studies with large population are required to demonstrate the incidence and the progress of PV stenosis after LAA occluder implantation.
